# The birth of an open access multidisciplinary online journal

**DOI:** 10.2349/biij.1.1.e1

**Published:** 2005-07-01

**Authors:** BJJ Abdullah, KH Ng

**Affiliations:** Department of Biomedical Imaging (Radiology), Faculty of Medicine, University of Malaya, Kuala Lumpur, Malaysia

Dear Colleagues,

We are proud to announce the birth of Biomedical Imaging and Intervention Journal (***biij***), an open access multidisciplinary online journal. ***biij*** was set up to meet the challenges of biomedical imaging and intervention facing the allied sciences community by providing a new avenue for discussion and exchange of viewpoints.

So how is ***biij*** different from other scientific online journals? ***biij***'s objectives are:

- to provide free online access to all our scientific materials in accordance with the Budapest Open Access Initiative. ***biij*** plans to remove unnecessary barriers that inhibit the sharing of scientific knowledge and information to accelerate research, enrich education and share the learning of the rich with the poor and vice-versa. ***biij*** lays the foundation to unite humanity in a common intellectual discussion and to fulfil the quest for knowledge in a cost-effective manner.
- to expand the scope of disseminating scientific information through the Internet by making available a large stock of teaching materials in the form of audio-video files from presentations made at scientific meetings.
- through shared ownership by the scholarly community and individuals involved in the field to shape the future direction of the journal including ‘radiological’ and allied organizations in the region that do not have their own journals to share their research publications, guidelines, etc. with others.

It gives us great pleasure to invite you to be a part of ***biij*** by contributing articles for publication. With your support, participation and commitment, we are confident that we can overcome challenges to make ***biij***, an open access multidisciplinary international online journal that will attract the best of publications in the field of biomedical imaging and intervention, a success.

Articles for publication can be in the form of:

- Original research
- Review articles
- Commentaries
- Teaching points
- ‘How I do it’
- Letters, correspondence, etc
- Case reports

We welcome your comments. Thank you.

